# Evolved distal tail protein of skunaviruses facilitates adsorption to exopolysaccharide-encoding lactococci

**DOI:** 10.20517/mrr.2023.29

**Published:** 2023-07-18

**Authors:** Anne M. Millen, Damian Magill, Dennis Romero, Laura Simdon

**Affiliations:** ^1^Health and Biosciences, IFF, Madison, WI 53716, USA.; ^2^Health and Biosciences, IFF, Dangé-Saint-Romain 86220, France.

**Keywords:** *Lactococcus*, *Skunavirus*, 936 group bacteriophage, exopolysaccharide, evoDit, dairy fermentations

## Abstract

**Aim:** Lactococcal skunaviruses are diverse and problematic in the industrial dairy environment. Host recognition involves the specific interaction of phage-encoded proteins with saccharidic host cell surface structures. Lactococcal plasmid pEPS6073 encodes genes required for the biosynthesis of a cell surface-associated exopolysaccharide (EPS), designated 6073-like. Here, the impact of this EPS on *Skunavirus* sensitivity was assessed.

**Methods:** Conjugal transfer of pEPS6073 into two model strains followed by phage plaque assays and adsorption assays were performed to assess its effect on phage sensitivity. Phage distal tail proteins were analyzed bioinformatically using HHpred and modeling with AlphaFold. Construction of recombinant phages carrying evolved Dits was performed by supplying a plasmid-encoded template for homologous recombination.

**Results:** pEPS6073 confers resistance against a subset of skunaviruses via adsorption inhibition. IFF collection skunaviruses that infect strains encoding the 6073-like *eps* gene cluster carry insertions in their distal tail protein-encoding (*dit*) genes that result in longer Dit proteins (so-called evolved Dits), which encode carbohydrate-binding domains. Three skunaviruses with classical Dits (no insertion) were unable to fully infect their hosts following the conjugal introduction of pEPS6073, showing reductions in both adsorption and efficiency of plaquing. Cloning the evolved Dit into these phages enabled full infectivity on their host strains, both wild type and transconjugant carrying pEPS6073, with recombinant phages adsorbing slightly better to the EPS^+^ host than wild type.

**Conclusion:** The 6073-like EPS potentially occludes the phage receptor for skunaviruses that encode a classical Dit protein. Skunaviruses that infect strains encoding the 6073-like EPS harbor evolved Dits, which likely help promote phage adsorption rather than just allow the phage to circumvent the putative EPS barrier. This work furthers our knowledge of phage-host interactions in *Lactococcus* and proposes a role for insertions in the Dit proteins of a subset of skunaviruses.

## INTRODUCTION

*Lactococcus lactis* and *Lactococcus cremoris* are mesophilic lactic acid bacteria used in starter cultures for dairy fermentations. The long-term utility of highly specialized starter strains is limited by their vulnerability to bacteriophages, including those belonging to the *Skunavirus* genus (936 group), which are commonly found in the industrial environment^[1,2]^. Phage infections can cause significant economic loss due to negative end-product quality issues and failed manufacturing processes. Extensive research into lactococcal phage-host interactions has identified a range of phage defenses that can be exploited in industrial strains for improved phage resistance^[3-5]^.

Cell wall polysaccharides (CWPS), encoded by the chromosomal *cwps* operon (also referred to as *rgp* operon), decorate the lactococcal cell wall and have been shown to serve as receptors for skunaviruses^[4,6-8]^. In the initial step of infection, a phage attaches to the cell by binding to its receptor. This attachment, known as phage adsorption, is mediated by phage adhesion modules, which include the C-terminus of the tail tape measure protein, the distal tail protein (Dit), the N-terminal region of the tail-associated lysin (Tal), the receptor binding protein (RBP), and baseplate proteins (BPPs), if present^[9-11]^. Dit is a major component of the *Skunavirus* baseplate, and two types of Dit proteins have been found to exist in skunaviruses: classical and long (evolved)^[8]^. Internal insertions are present in the evolved Dits (evoDits), with the N‐terminal and C‐terminal regions aligning with the full length of the classical Dit. These insertions were found to encode carbohydrate-binding modules (CBMs) and were shown to promote host-binding^[8]^. In addition to *Lactococcus*, evoDits have been shown to be involved in phage adsorption in lactobacilli and *Bacillus cereus*^[12]^. Analyses showed the Dit-associated CBMs of lactococcal skunaviruses exhibit the same strain specificity as the RBPs and likely recognize a saccharidic component of the CWPS^[8]^. Indeed, a Dit/RBP/CWPS correlation could be made across subtypes within the *Skunavirus* genus^[8]^. Lactococcal evoDits were previously grouped into four classes^[8]^. Subsequently, an analysis of IFF (formerly DuPont) collection skunaviruses identified as many as 16 distinct groups of Dit insertions^[4]^.

In addition to CWPS, some lactococci also produce exopolysaccharides (EPS), which may be excreted into the growth media or tightly associated with the cell surface^[13]^. EPS-producing strains are valuable for the desirable textures they produce in fermented dairy products^[13]^. Additionally, plasmid-encoded EPS biosynthetic capability has been demonstrated to reduce the adsorption of certain phages to the cell, increasing the phage robustness of the strain^[14-16]^. However, EPS-mediated phage resistance appears to be limited, as many examples of phages infecting EPS-producing lactococcal strains have been reported^[17]^. Furthermore, we recently identified two subsets of P335-group phages, each of which differentially adsorbed to strains that encode a distinct set of EPS-associated gene clusters^[18]^. These gene clusters, originally identified on plasmids pEPS6073 and pEPS7127, were designated 6073-like and 7127-like, with further designation of a 6073-like variant (EpsM variant). The 6073-like and 7127-like *eps* gene clusters were each found to encode genes required for the synthesis of a cell surface-associated EPS, which, we proposed, functions as a receptor for the respective P335 phages^[18]^.

In this study, the work was extended to another problematic phage group, and pEPS6073 was found to provide resistance against a subset of skunaviruses via adsorption inhibition. The IFF phage collection, however, contains several skunaviruses that infect hosts known to harbor the 6073-like *eps* gene cluster, indicating this resistance does not extend across the entire *Skunavirus* genus. Here the genetic determinants enabling phages to infect 6073-like EPS^+^ strains were investigated. Skunaviruses that infect hosts encoding the 6073-like *eps* gene cluster were found to carry insertions in their distal tail proteins, which encode carbohydrate-binding domains. The relationship between the Dit insertion and EPS^+^ host was confirmed through the construction of recombinant phages carrying Dit insertions. This work furthers our knowledge of phage-host interactions in *Lactococcus* and proposes a role for insertions in the Dit proteins of certain skunaviruses.

## METHODS

### Bacterial strains and phages

Bacterial strains and phages are listed in Table 1. Lactococcal strains were grown at 30 °C in sterile 11% w/v nonfat dry milk (NFDM) or in M17 broth (Oxoid, UK) supplemented with 0.5% lactose or glucose. *Escherichia coli* was aerobically propagated in LB broth (BD Difco, USA) at 37 °C. When required, antibiotics were added to the media as follows: erythromycin [Em; 5 µg/mL (lactococci), 150 µg/mL (*E. coli*)].

**Table 1:** Strains, bacteriophages, and plasmids

**Biological material**	**Relevant characteristics**	**Reference**
**Bacteria**		
*Lactococcus lactis*		
1403S	Spontaneous Sm^R^ derivative of IL1403, plasmid-free	[19]
1403S-EPS	Sm^R^, Em^R^, pEPS6073^+^	[18]
1403S-Dit_6887_	1403S + pG9Dit_6887_	This study
*Lactococcus cremoris*		
DGCC6071	Starter strain 6073-like EPS EpsM var.	This study
DGCC6073	Starter strain 6073-like EPS typical	[18]
DGCC6871	Starter strain 6073-like EPS EpsM var.	This study
DGCC7168	Starter strain 6073-like EPS EpsM var.	This study
DGCC7193	Starter strain 6073-like EPS EpsM var.	This study
DGCC8692	Starter strain 6073-like EPS typical	This study
LM2345	Plasmid-free, Sp^R^	[20]
2345-EPS	Sp^R^, pEPS6073^+^	[18]
2345-Dit_6887_	LM2345 + pG9Dit_6887_	This study
*Escherichia coli*		
TG1 RepA^+^	*SupE hsd*∆*5 thi* ∆(*kac-proAB*) F’ (*traD36 proAB*^+^ *lac*I^q^ *lacZ*∆M15) with chromosomal copy of pWV01 *repA*	[21]
**Bacteriophages**		
D753	Host DGCC6871	This study
D970	Host DGCC8692	This study
D1113	Host DGCC8692	This study
D2929	Host DGCC7168	This study
D4006	Host DGCC7193	This study
D4839	Host DGCC6073	This study
D4842	Host DGCC6071	This study
D5604	Host DGCC6071	This study
D6067	Host DGCC8692	This study
D6869	Host DGCC6073	This study
D6875	Host DGCC6073	This study
D6887	Host DGCC6073	This study
D6888	Host DGCC6073	This study
p2	Host LM2345	[22]
bIL170	Host 1403S	[23]
P008NC	Host 1403S; derivative of P008^[24]^	North Carolina State collection
p2-Dit_6887_	p2 encoding Dit_6887_	This study
bIL170-Dit_6887_	bIL170 encoding Dit_6887_	This study
P008NC-Dit_6887_	P008NC encoding Dit_6887_	This study
**Plasmids**		
pGhost9	Em^R^, temperature sensitive vector	[25]
pG9Dit_6887_	pGhost9 + Dit_6887_	This study

Dit: Distal tail protein; Em^R^: erythromycin-resistant; EPS: exopolysaccharide; Sm^R^: streptomycin-resistant; Sp^R^: spectinomycin-resistant; var.: variant.

The preparation of bacteriophage lysates was performed as previously described^[26]^. High titer lysates were passed through a 0.45 µm filter and stored at 4 °C. Phage typing was performed using the multiplex PCR method as described by Labrie and Moineau^[27]^. Spot titer assays and plaque assays were performed as previously described^[26]^ on MRS medium (Oxoid, UK). Briefly, 10-fold serial dilutions in peptone buffer (3M, USA) of phage lysates were prepared. For standard plaque assays, 100 µL of the phage dilution was combined with 150-200 µL of exponentially growing cells (A_600_ ≈ 0.5) and incubated at room temperature for 10 min. The mixture was added to 3 mL of soft agar overlay (0.5% agar w/v) containing 10 mM CaCl_2_ and then poured onto a standard MRS agar base plate. For spot assays, exponentially growing cells were added to the soft agar overlay with CaCl_2_ and then poured onto a base plate. Once solidified, aliquots of the 10-fold serially diluted phage lysates were spotted onto plates. The Efficiency of Plaquing (EOP) is determined by dividing the phage titer on the test host by the titer on the fully sensitive host strain. Results are averaged from three independent replicates.

Phage adsorption tests were performed as previously described^[28]^ on MRS medium. Briefly, phage and exponentially growing cells (absorbance at 600 nm = 0.5) were combined and incubated for 15 min at room temperature. The mixture was then centrifuged (RCF = 9,750 × g at 4 °C) and supernatant assayed for residual phage by standard plaque assay on a sensitive host. Percent adsorption is calculated as: (starting phage titer - residual phage titer)/starting phage titer, and then multiplied by 100. Adsorption on the sensitive host serves as the control against which test strains are compared. Results are averaged from three independent replicates.

### PCR, DNA preparation, sequencing, and bioinformatic tools

PCRs were performed with GoTaq® Colorless Master Mix (Promega Corp., USA) or Phusion HF Mastermix (Thermo Scientific, USA) according to the manufacturer’s instructions with 35 cycles of denaturation/annealing/extension. Primer sequences and additional thermocycler conditions are listed in Table 2. Amplicons were purified using the Promega Wizard® SV Gel and PCR Clean-Up System (Promega Corp., USA). Primers were synthesized by Integrated DNA Technologies (USA). Phage DNA was isolated from high-titer phage lysate using Invitrogen PureLink Viral RNA/DNA Mini kit (Life Technologies, USA) according to the manufacturer’s instructions. Phage DNA was sequenced using Illumina technology (Illumina, USA). Sanger sequencing was performed by Eurofins Genomics (USA). Sequences were annotated using RAST or PATRIC RASTtk-enabled Genome Annotation Service^[29-31]^. Sequences were analyzed using Geneious Prime 2021.0.3 (https://www.geneious.com). Protein analysis was performed using HHpred (Homology detection & structure prediction by HMM-HMM comparison)^[32,33]^.

**Table 2:** Primer sequences and thermocycler conditions

**Primer name**	**Sequence (5’ - 3’)**	**Thermocycler conditions**
Clone 6073DIT-F	TTCTGGAATGGGATTCAGGACCTAGGGAGGGCTTAATGG	Annealing temperature: 57 °C/extension time: 45 s
Clone 6073DIT-R	ATCCGTGTCGTTCTGTCCACCATTAAACGAAGTCCGCCTTTC	
VF-pG9	GTGGACAGAACGACACGGAT	Annealing temperature: 55 °C/extension time: 1 min 45 s
VR-pG9	TCCTGAATCCCATTCCAGAA	
CheckDIT-F	CTAGCGGTTACGGTTTAAGC	Annealing temperature: 53 °C/extension time: 1.5 min
CheckDIT-R	CCCAYARTTCATARTTAATRAC	
p2RPB-F	TGTTAGAGCTATCAATTACTG	Annealing temperature: 53 °C/extension time: 30 s
p2RPB-R	CCATGTTGCGAA AAGAATCG	

### Molecular cloning and phage engineering

Development of pG9Dit_6887_ was performed using NEBuilder® HiFi DNA Assembly (New England Biolabs, USA). PCR amplification of *dit* was conducted from D6887 phage lysate with primers clone 6073DIT-F and clone 6073DIT-R [Table 2]. A linear amplicon of pGhost9, a vector commonly used for cloning experiments, was created by PCR with primers VF-pG9 and VR-pG9 [Table 2]. Purified Dit amplicon was assembled with purified linear pGhost9 using NEBuilder® HiFi DNA Assembly Master Mix (New England Biolabs, USA) according to the manufacturer’s instructions. Assembly was electroporated into *E. coli* TG1 RepA^+^ cells according to the Dower method^[34]^. Recombinant plasmids were isolated from TG1 RepA^+^ cells using GeneJET Plasmid Miniprep Kit (Thermo Scientific, USA). Purified plasmid was electroporated into lactococci as previously described^[35]^.

To isolate recombinant phages that have exchanged the wild-type *dit* for *dit*_6887_, phages were propagated on their respective lactococcal host containing pG9Dit_6887_. With sufficient DNA homology between the respective 5’ and 3’ ends of the wild-type *dits* and *dit*_6887_, recombination between the phages and pG9Dit_6887_ would occur via double crossover [Supplementary Figure 1]. Selection of recombinant phages was performed by plating the propagations on the respective host + pEPS6073.

### Molecular modeling

Molecular modeling of Dit proteins was conducted using the AlphaFold package in monomer mode and employing the fully compiled database of Protein Data Bank (PDB) structures^[36]^. Predictions were scored based on the pLDDT criteria in order to choose the top-ranked model. Putative carbohydrate-binding domains were predicted using an in-house software package. Structural alignments were carried out using the TM-align algorithm^[37]^. Visualization of models and preparation of publication-grade figures was conducted within Pymol^[38]^.

### Statistical analysis

One-tailed *t*-tests were conducted for the comparison of adsorption data across recombinant and native phages on the relevant strains. This was carried out using the SciPy package within Python.

## RESULTS

### pEPS6073 confers resistance against skunaviruses in model lactococci

Lactococcal plasmid pEPS6073 encodes a previously characterized cell surface-associated exopolysaccharide (EPS) correlated to sensitivity to a subgroup of P335 phages^[18]^. Because plasmid-encoded EPS has been shown to provide phage resistance in lactococci by reducing phage adsorption^[14]^, *L. cremoris* LM2345 and *L. lactis* 1403S transconjugants containing pEPS6073 (2345-EPS and 1403S-EPS) were tested against homologous skunaviruses. Plaque assays, which measure the level of phages present that are able to lyse the cell, showed pEPS6073 provided a high level of resistance to each phage as evidenced by a low efficiency of plaquing (EOP) [Table 3]. Adsorption assays, which measure the level of phages that are able to attach to the cells, showed a reduction in adsorption of each phage to the EPS^+^ transconjugant compared to the fully sensitive parent strain [Table 3].

**Table 3:** Introduction of pEPS6073 into lactococcal host strains provides resistance against select skunaviruses

**Phage**	**Strain**	**EOP**	**Adsorption (%)**	**Comments**
p2	LM2345	1	89.3 ± 2.1	Clear plaques
	2345-EPS	3.4 × 10^-6^ (± 3.3 × 10^-6^)	26.0 ± 2.3	Turbid plaques
bIL170	IL1403S	1	97.7 ± 1.2	-
	1403S-EPS	< 2.9 × 10^-9^	43.9 ± 16.6	No plaquing, poor bacterial lawn at highest phage titers
P008NC	IL1403S	1	95.0 ± 0.9	-
	1403S-EPS	< 1.2 × 10^-9^	47.7 ± 10.5	No plaquing, poor bacterial lawn/clear at the highest phage titers

Phage plaque and adsorption assays were carried out on model lactococcal strains +/- pEPS6073. When applicable, the EOP is the average of three independent trials ± sample standard deviation. When no plaques were visible, EOP is < the highest value of three independent trials. Adsorption is the average of three independent trials ± sample standard deviation. EOP: Efficiency of plaquing.

### Skunaviruses that infect 6073-like EPS^+^ strains contain insertions in Dit

**Figure 1:**
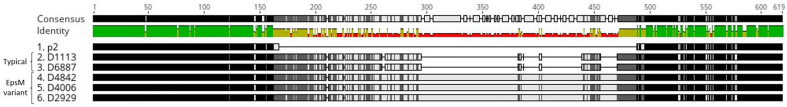
Amino acid alignment of representative Dits. Deduced amino acid alignment of the classical Dit from phage p2 and the evolved Dits from representative phages that infect hosts encoding a 6073-like *eps* gene cluster. Phages that infect strains encoding the typical 6073-like EPS *vs.* EpsM variant are indicated. In the consensus identity bar, green indicates identity, yellow indicates polymorphism, and red indicates low identity. Dit: distal tail protein; EPS: distal tail protein; EpsM: 6073-like variant.

The IFF collection includes skunaviruses that infect strains, including *L. cremoris* DGCC6073, that encode the 6073-like *eps* gene cluster. To determine why these phages are able to infect EPS^+^ hosts, while pEPS6073 provides a level of resistance against p2, bIL170, and P008NC (a derivative of P008 received from North Carolina State University that shares 97% nucleotide identity with P008), whole genome sequencing was performed on five skunaviruses that infect DGCC6073 and two that infect *L. cremoris* DGCC6071, a strain that encodes the EpsM variant of the 6073-like *eps* gene cluster. Examination of the genes encoding the phage distal tail proteins (Dits) found that the skunaviruses infecting DGCC6073 and DGCC6071 contain evolved Dits, which harbor insertions encoding carbohydrate-binding domains. The Dits of the phages infecting DGCC6073 (D4839, D6869, D6875, D6887, and D6888) share > 97% deduced amino acid identity and contain inserts of 168 aa [Supplementary Table 1 and Figure 1]. This Dit insert is consistent with group 14, based on our previous analysis of IFF collection skunaviruses that identified 16 distinct groups of Dit insertions^[4]^. The Dits of the phages infecting DGCC6071 (D4842 and D5604) share 100% deduced amino acid identity and contain inserts of 319 aa [Supplementary Table 1 and Figure 1], which are consistent with group 7. HHpred analyses of these Dit insertions result in top domain hit 5E7T_B; minor structural protein 5; bacteriophages; *Lactococcus lactis*. Minor structural protein 5 is identified as ORF 52/BppA in lactococcal phage TUC2009 (accession number NC_002703), a component of the tripod baseplate structure with a putative carbohydrate-binding domain and is involved in receptor binding^[39,40]^. We observed the presence of sequences in the public domain similar to our group 7 classified Dits, with an average identity of 55%. Through HHpred analysis, the proteins from these phages (62601, 62605, and CHPC958) were also found to be in possession of the 5E7T_B domain.

**Figure 2:**
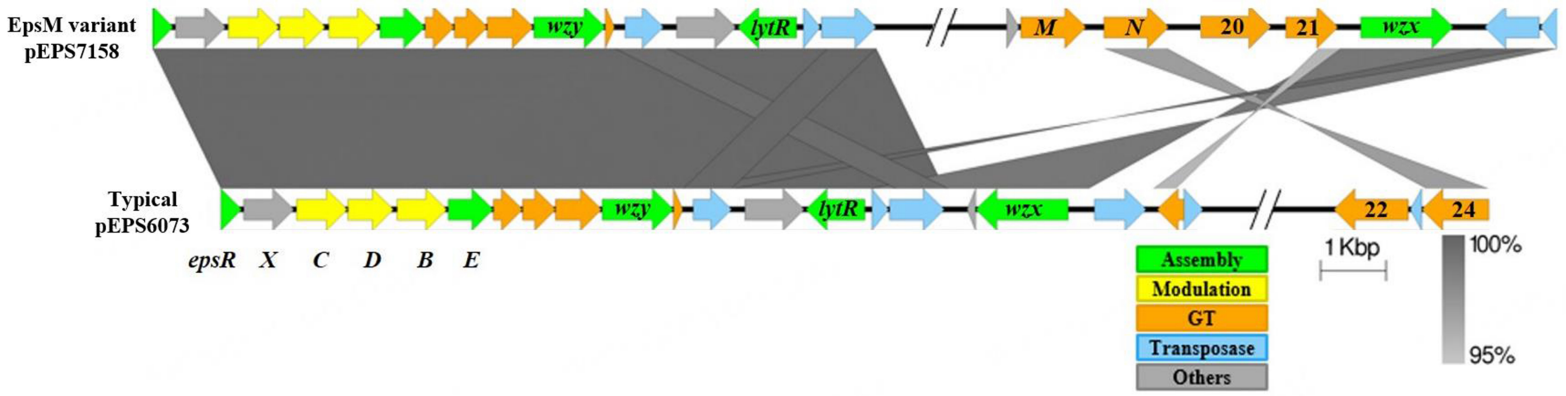
Alignment of typical and EpsM variant *eps* gene clusters as they reside on pEPS6073 and pEPS7158, respectively (Excerpted from Millen *et al.*, 2022^[18]^). Numbers appearing over gene depictions correspond to locus tags as annotated in GenBank (accession numbers OP323065-OP323068). Genes are colored based on their putative function, including EPS assembly, EPS modulation, glycosyltransferase, transposase, or others. The putative polymerase (*wzy*), flippase (*wzx*), and attachment (*lytR*) genes are annotated as such. Gray bars are used to indicate sequence identity. EPS: Distal tail protein; EpsM: 6073-like variant.

Additional sequenced IFF collection phages that encode Dits sharing high nucleotide identity to those present in phages infecting DGCC6073 or DGCC6071 were identified (representative translated Dits shown in Figure 1). Each of these phages was found to infect a host that encodes a 6073-like *eps* gene cluster. Furthermore, the specific Dit insertion was found to correlate to the subtype of the host-encoded 6073-like *eps* gene cluster: typical (as described on pEPS6073) or the closely related EpsM variant^[18]^. We previously described the EpsM variant as it resides on pEPS7158^[18]^, and an alignment of the typical *vs.* EpsM variant *eps* clusters as they reside on respective plasmids pEPS6073 and pEPS7158 extracted from Millen *et al.*, 2022^[18]^ can be found in Figure 2. The EpsM variant can be distinguished from the typical 6073-like cluster by differences in a set of two glycosyltransferase (GTF) genes found outside of the contiguous *eps* operon. pEPS6073 contains GTF genes pEPS6073_22 and pEPS6073_24, whereas pEPS7158 contains GTF genes annotated as *epsM* and *epsN*^[18]^. Since GTFs are responsible for linking the sugars of the EPS, and each GTF may have a different substrate specificity^[41]^, the differences in GTF content between the 6073-like strain and its variant likely affect the composition of the EPS.

**Figure 3:**
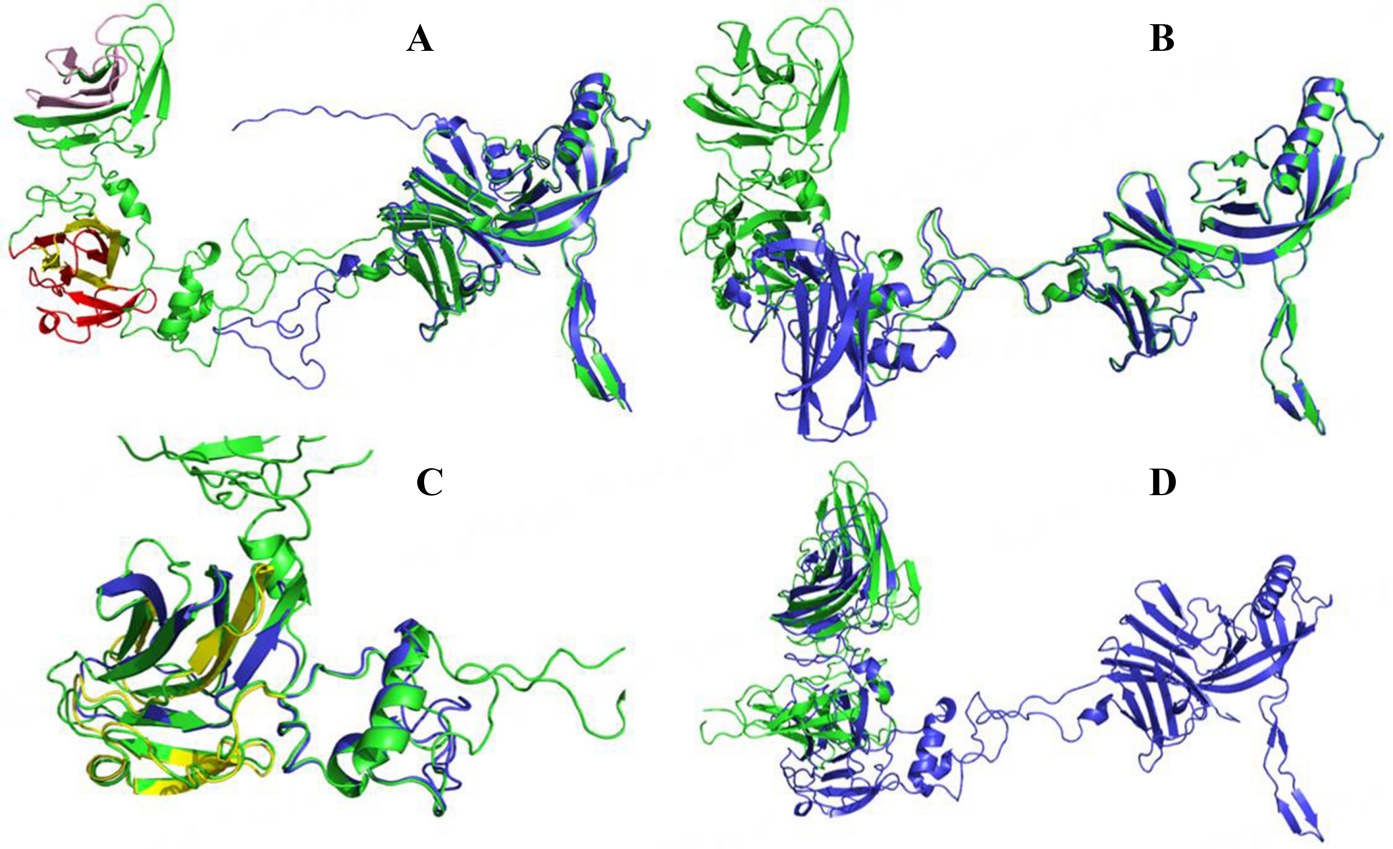
Molecular modeling and comparison of select classical and evolved Dit proteins. Structural alignment of the AlphaFold predicted Dit models for phages D2929 (green) and M7361 (blue), respectively (image A). The region corresponding to the insert present in D2929 is shown on the left-hand side of the figure, with the locations of the three predicted carbohydrate-binding regions (two of them corresponding to the proximal domain and one corresponding to the distal domain) highlighted in different colors. Structural alignment of the D2929 (green) and D6887 (blue) Dit models is provided in panel B on the right-hand side of the figure with an alignment corresponding only to the insert regions provided in panel C at the bottom. The predicted carbohydrate-binding motif in D6887 is highlighted in yellow. Panel D at the bottom right shows a structural alignment of the D4006 Dit protein predicted to possess BppA domains aligned with the minor structural protein 5 (BppA) from the TUC2009 solved baseplate structure (PDB: 5E7T). It can be clearly seen in the proximal portion of the insert in D4006 that there is a structural similarity with BppA. BppA: accessory base plate protein; Dit: distal tail protein; PDB: protein data bank.

Dits from phages D970, D1113, and D6067, which all infect *L. cremoris* DGCC8692 (encoding a typical 6073-like *eps* gene cluster), are ~99% identical at the deduced amino acid level and share 91%-94% pairwise amino acid identity with the Dits of the phages infecting DGCC6073 [Supplementary Table 1 and Figure 1]. The Dits of phages D753, D4006 and D4842, each of which infects a different *L. cremoris* strain that harbors the EpsM variant 6073-like *eps* gene cluster (DGCC6871, DGCC7193, or DGCC6071, respectively), share > 99% deduced amino acid identity [Supplementary Table 1]; however, the Dit of D2929, also infecting an *L. cremoris* strain encoding the EpsM variant EPS (DGCC7168), shares only ~87% deduced amino acid identity with the Dit of the other three phages [Supplementary Table 1 and Figure 1]. Notably, Dits of the phages that infect strains encoding a typical 6073-like EPS share only 58%-62% pairwise amino acid identity with those of the phages that infect strains encoding the EpsM variant EPS. Furthermore, HHpred analyses found that the Dit insertions of the phages infecting hosts that encode the EpsM variant *eps* gene cluster contain two 5E7T_B domains compared to the single domain encoded by the insertions in the Dits belonging to phages that infect hosts harboring the typical 6073-like *eps* gene cluster. Representative evolved Dit from D2929 was modeled and compared with the classical Dit from M7361^[4]^ as well as with D6887 [Figure 3]. A clear conservation of the core region from the classical Dit compared to the evolved counterparts can be observed, with the inserts existing as a distinct region connected to the core region via a series of flexible loops. This highlights a modular evolution within the protein, further exemplified when comparing D6887 with D2929, the latter containing two distinctly visible domains within the insert region. Both domains within the D2929 insert region were predicted to contain motifs involved in carbohydrate binding, supporting the HHpred results. The proximal domain was predicted to contain two contiguous regions located at residues 188-259 and 280-321 involved in carbohydrate binding, whereas the distal domain was predicted to contain one such motif across the amino acid range 391-432. With respect to D6887, the absence of the D2929 distal domain is immediately apparent in the structure. Alignments of the proximal domain revealed a 92% structural similarity; however, it was observed that in the case of D6887, only one carbohydrate-binding motif was predicted, corresponding to the one located in the range 188-259 in D2929. The presence of BppA-like domains within the Dits of some phages also represented a point of interest, as the accessory base plate protein (BppA) has been shown to contain CBMs involved in phage binding to the host^[8,40,42]^. Therefore, a model was constructed of the Dit from phage D4006, predicted to contain this domain, and was compared to the solved structure for TUC2009 (5E7T_B) [Figure 3D]. Indeed, it was observed that there exists a structural similarity between the proximal portion of the insert in the D4006 Dit and 5E7T_B.

### Development of recombinant phages

To determine if Dit insertions are necessary for *Skunavirus* virulence on 6073-like EPS^+^ strains, three model phages that contain classical Dits (bIL170, P008NC, and p2) were engineered to encode the Dit insertion from D6887. Vector pG9Dit_6887_, which includes the entire *dit* sequence from D6887, was assembled in *E. coli* TG1 RepA^+^, and the constructed plasmid was then introduced into 1403S and LM2345. Phages p2, P008NC, and bIL170 were propagated on their respective host containing pG9Dit_6887_ until the culture cleared from phage lysis. Phage lysates were then plaqued on their respective hosts containing pEPS6073. Although pEPS6073 provides a level of resistance to the wild-type phages, titers of these phage propagations on their respective pEPS6073^+^ host strains were > 1 × 10^6^ pfu/mL with clear plaque morphology, indicating a high level of phages within each of the respective lysates was not inhibited by pEPS6073. A representative single plaque isolate from each of the three plaque assays was propagated on its respective host containing pEPS6073. PCR analysis of each lysate with primers checkDIT-F and checkDIT-R showed an increase in *dit* size, indicative of an exchange of the D6887 Dit with the native Dits of phages p2, bIL170, and P008NC. Illumina sequencing of recombinant phages p2-Dit_6887,_ bIL170-Dit_6887_, and P008NC-Dit_6887_ confirmed the successful integrations of the Dit_6887_ insertion. Besides the Dit_6887_, no differences between the bIL170-Dit_6887_ genome and the published bIL170 genome (AF009630) were found. In addition to the Dit_6887_, only one SNP (silent mutation in the 3’ end of the putative neck passage structure CDS) was observed in P008NC-Dit_6887_ compared to the sequence of the P008NC phage lysate used for its development. Three additional SNPs were observed between the p2-Dit_6887_ genome and the published p2 genome (NC_042024); one intergenic, one silent, and one in the receptor-binding protein (RBP). All three loci in the p2 lysate used for the development of the recombinant phage were confirmed to be consistent with the published genome. As the receptor binding protein is the primary phage antireceptor, the RBP was sequenced in three additional p2-Dit_6887_ isolates using primers p2RPB-F and p2RPB-R. No mutations were identified in the RBP of these recombinant phage isolates, confirming that RBP mutation is not required for infection on the EPS^+^ host.

**Figure 4:**
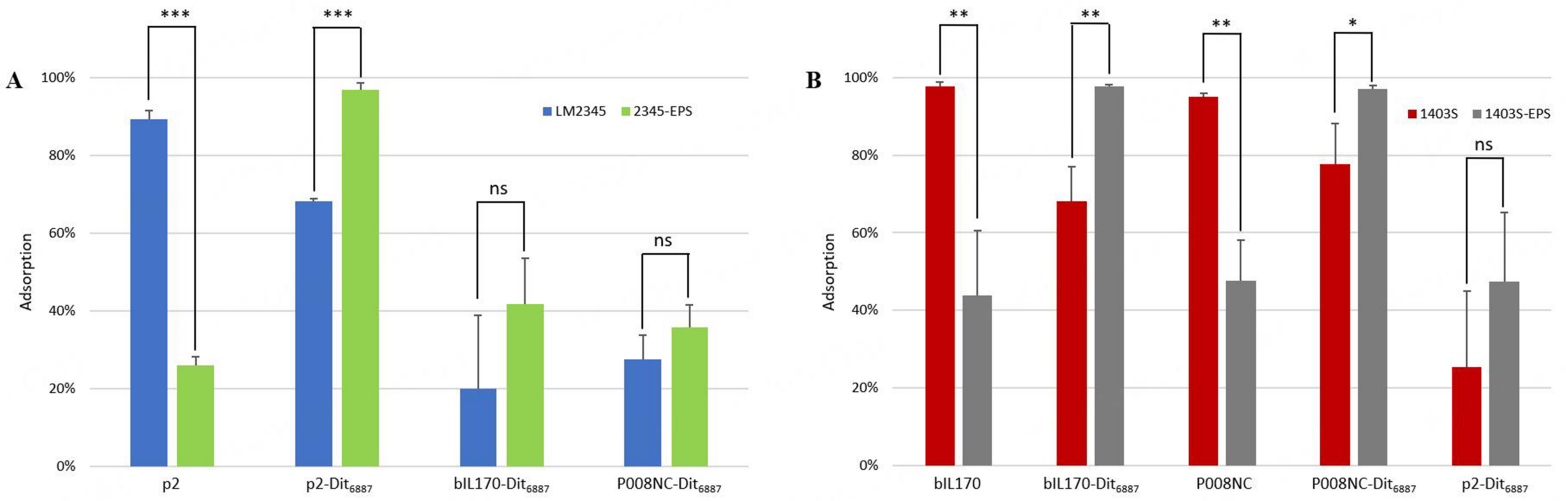
Dit promotes phage adsorption to EPS^+^ strains. (A): Assays on LM2345 and EPS^+^ transconjugant; (B): Assays on 1403S and EPS^+^ transconjugant. Adsorption assays were performed with wild-type and recombinant phages on isogenic EPS +/- strains. Wild-type phages adsorbed strongly to their native host, and the introduction of pEPS6073 decreased their adsorption (*P* < 0.05). Dit exchange resulted in poorer adsorption to each native host but strong adsorption to the pEPS6073^+^ transconjugant of the native host (*P* < 0.05). Recombinant phages adsorbed poorly to non-native hosts, although adsorption was slightly increased on the non-native host when pEPS6073 was present (not statistically significant). Statistical comparisons conducted to support the various conclusions have been annotated using connecting lines to explicitly show the pairwise calculations performed. Statistically significant comparisons are marked with asterisks that indicate the level of statistical significance according to the following scale: (* *P* < 0.05, ** *P* < 0.01, *** *P* < 0.001). Comparisons that are not statistically significant are labeled ns. Wild-type phages were not tested against non-native host strains. Average of three independent trials. Dit: Distal tail protein; Error bars: sample standard deviation; EPS: exopolysaccharides.

Recombinant phages were plaque assayed on their respective hosts +/- pEPS6073 [Table 4]. The EOPs of P008NC-Dit_6887_ were roughly equivalent on both isogenic hosts. The EOPs of bIL170-Dit_6887_ and p2-Dit_6887_ were both slightly reduced on the pEPS6073^-^ host (< 1 log EOP reduction); however, plaque sizes of all recombinant phages were slightly reduced on the pEPS6073^+^ host. This data shows that recombinant phages can infect both their EPS^-^, wild-type host and the respective isogenic EPS^+^ transconjugant. To determine how the exchanged Dit_6887_ affects phage adsorption, adsorption assays were performed with P008NC-Dit_6887_, bIL170-Dit_6887_, and p2-Dit_6887_ [Figure 4]. Wild-type phages were only tested on their homologous (fully infective) hosts, but recombinant phages were also tested on non-homologous hosts to determine if the EPS facilitates adsorption when the evolved Dit is present. Recombinant phages showed higher adsorption to their EPS^+^ host than the EPS^-^ isogenic host. Additionally, recombinant phage adsorption to the EPS^-^ host was reduced compared to that of the wild-type phages. Assays of the recombinant phages on the non-homologous host (p2/1403S, bIL170/LM2345, and P008NC/LM2345) did not show a strong increase in adsorption to the EPS^+^ strain compared to the EPS^-^ isogenic strain, indicating that the presence of the EPS alone is not sufficient for efficient adsorption [Figure 4].

**Table 4:** Recombinant phages show expanded host range

	**EOP**				**Plaque size (mm)**			
	1403S	1403S-EPS	LM2345	2345-EPS	1403S	1403S-EPS	LM2345	2345-EPS
P008NC-Dit_6887_	1.08 ± 0.19	1	NA	NA	1.75 ± 0.17	1.49 ± 0.04	NA	NA
bIL170-Dit_6887_	0.36 ± 0.11	1	NA	NA	1.54 ± 0.10	0.84 ± 0.24	NA	NA
p2-Dit_6887_	NA	NA	0.47 ± 0.46	1	NA	NA	0.64 ± 0.21	0.54 ± 0.08

Plaque assays were performed with recombinant phages on their respective hosts +/- pEPS6073. Average of three independent trials. Three plaques were measured for each assay. Error bars: Sample standard deviation. EOP: efficiency of plaquing. NA: not applicable as the strain is not a homologous host for the respective phage.

## DISCUSSION

We recently described lactococcal *eps* gene clusters that are each associated with sensitivity to a subgroup of P335 phages^[18]^. One such cluster, designed 6073-like, was originally identified on pEPS6073, and a closely related variant (EpsM variant) with differences in a set of two glycosyltransferases (GTFs) found outside of the contiguous *eps* operon was subsequently identified^[18]^. Despite its previous association with sensitivity to a P335 group phage subset, pEPS6073 was found to provide resistance to a subset of skunaviruses in model lactococcal strains. Upon conjugal introduction, pEPS6073 reduced the adsorption of skunaviruses bIL170, P008NC, and p2 to their model host transconjugants. Therefore, adsorption inhibition, at least in part, accounted for this phage resistance phenotype. The majority of the 34.4 kb pEPS6073 encodes the *eps* gene cluster, mobile elements, and replication functions; however, there is roughly 8 kb of the plasmid that encodes several hypothetical proteins (data not shown). Therefore, it cannot be ruled out that other genes encoded on pEPS6073 could play a role in phage resistance. However, our data are consistent with previous reports of plasmid-encoded EPS providing phage adsorption inhibition and resistance in lactococci^[14,16]^. This adsorption inhibition is proposed to result from the cell surface-associated EPS occluding the saccharidic cell surface receptor required by the phages^[14,16]^. Our results are aligned with this proposal, as we have previously shown via electron microscopy that the EPS produced by pEPS6073 is associated with the cell surface^[18]^. We found that this putative 6073-like EPS-associated phage resistance does not apply to all skunaviruses, as the IFF phage collection contains several that infect strains encoding a 6073-like *eps* gene cluster (either typical or EpsM variant), including DGCC6073, the native host of pEPS6073. Therefore, we sought to understand why the 6073-like EPS may provide resistance to some, but not all, skunaviruses.

Available genomes of IFF collection skunaviruses that infect 6073-like EPS^+^ strains were compared to those of bIL170, P008NC, and p2. Noticeably, phages that infect EPS^+^ strains had insertions in their distal tail proteins, consistent with an evolved Dit. Furthermore, the specific insertion could be correlated to the subtype of the 6073-like *eps* cluster encoded by the host, typical *vs.* EpsM variant. This was notable as Dit is a component of the *Skunavirus* baseplate, which is involved in phage-host interactions^[9]^. Classical and long (evolved) Dits have been previously described in skunaviruses, with internal insertions present in the evolved Dits found to contain carbohydrate-binding modules (CBMs)^[4,8]^. Likewise, the Dit inserts identified in this study were also predicted to contain CBMs, which is consistent with the evolved Dits potentially interacting with an EPS. Notably, HHpred analyses identified that the Dit insertions of the phages that infect hosts encoding the typical 6073-like *eps* gene cluster harbor a single 5E7T_B CBM, while Dit insertions of phages that infect hosts encoding the EpsM variant *eps* gene cluster contain two 5E7T_B domains. Modeling of representative Dits using AlphaFold further predicted two carbohydrate-binding domains in the D2929 Dit insert, with the proximal domain containing two contiguous regions involved in carbohydrate binding and the distal domain containing only one. In contrast, the Dit insert of D6887 lacked the distal domain and was predicted to encode only one carbohydrate-binding motif in the proximal domain. These differences likely account for the correlation observed between Dit insert and host EPS subtype. We previously showed that despite the differences in GTF content between the 6073-like EPS subtypes, strains encoding either EPS subtype adsorbed a specific subset of P335 group phages at high efficiency^[18]^. In contrast, this study would suggest that the differences between the EPS produced by the 6073-like *eps* gene cluster subtypes affect *Skunavirus* adsorption.

The evolved Dit encoded by D6887 was cloned into phages bIL170, P008NC, and p2. Recombinant phages were tested on their respective isogenic EPS^-^ wild-type and pEPS6073^+^ transconjugant hosts. While wild-type phages were inhibited on their pEPS6073^+^ host, displaying hazy plaques and/or reduced EOPs, all recombinant phages formed clear plaques on both wild-type and pEPS6073^+^ hosts, although plaque size was slightly reduced on EPS^+^ strains. Recombinant phages were found to plaque with comparable efficiencies on both their EPS^+^ and EPS^-^ hosts, indicating that the Dit insertion is indeed responsible for overcoming pEPS6073-mediated phage resistance. Furthermore, this demonstrated that the acquisition of the evolved Dit results in an expansion of the host range.

Because the pEPS6073-mediated phage resistance was shown to result, at least in part, from phage adsorption inhibition, adsorption assays were performed using recombinant phages. These assays found that the recombinant phages were able to adsorb at high efficiency to their EPS^+^ isogenic host, confirming that the Dit insertion enabled the phages to overcome the putative EPS-mediated adsorption inhibition. Adsorption assays were also used to determine if the Dit insertion facilitates phage adsorption rather than just enables the phages to circumvent the putative EPS barrier. Assays found that the adsorption of the recombinant phages to the EPS^-^ host was reduced compared to the adsorption of the wild-type phages, indicating that the recombinant Dit is less suited to interact with the wild-type, EPS^-^ host receptors. Assays also found that the recombinant phages adsorb to the EPS^+^ host with higher efficiency than the EPS^-^ isogenic host, indicating that the Dit insertion may facilitate adsorption to the putative EPS receptor. However, this is somewhat inconsistent with the adsorption assay results of the recombinant phages on the non-permissive host, which showed only a slight increase in adsorption to the EPS^+^ strain vs the EPS^-^ isogenic strain. The low efficiency of recombinant phage adsorption to the EPS^+^ non-permissive host could be due to incompatibilities of the strains’ cell wall polysaccharides (CWPS), which serve as a primary receptor for skunaviruses^[7,8]^; LM2345 encodes a type C.1 CWPS, while 1403S encodes a type B. This shows that while Dit helps facilitate adsorption to EPS^+^ strains, other phage and host factors are likely required for efficient adsorption, including the primary receptor/antireceptor (host CWPS/phage-encoded receptor-binding protein^[43,44]^). CBMs have also been found to decorate various phage structural components in addition to Dit, including the neck passage structures and major tail proteins of skunaviruses^[42]^. Together, these CBMs were proposed to contribute to phage adsorption by allowing more flexibility in binding orientation or by increasing host binding affinity due to their avidity^[42]^.

This study demonstrates that, like other previously described plasmid-encoded EPS, pEPS6073 reduces adsorption and provides resistance against a subset of skunaviruses. Coupled with our previous study that implicates the same plasmid-encoded EPS in P335 phage sensitivity^[18]^, this work shows the acquisition of EPS can alter a strain’s spectrum of phage sensitivity, providing resistance to some phages but acquiring sensitivity to others. This study found that skunaviruses that were inhibited by pEPS6073 encode classical Dits, while skunaviruses capable of infecting 6073-like EPS^+^ hosts contain insertions in their Dits that encode carbohydrate-binding domains. Such domains were previously shown to be involved in the host attachment of skunaviruses, likely recognizing a component of the CWPS^[8]^. This study indicates that a component of EPS is likely recognized by the evoDits encoded by strains that produce a 6073-like EPS, as recombinant phages with Dit_6887_ show increased adsorption to strains encoding the EPS. However, the EPS is not required for the adsorption of these phages, nor is the EPS alone sufficient to achieve a high level of phage adsorption, indicating additional phage/host factors are required for successful host attachment. This study increases our understanding of phage-host interactions regarding lactococcal EPS, which can be applied to the development of improved dairy starter cultures with increased phage robustness.

## DECLARATIONS

### Availability of data and materials

Representative evolved Dit sequences presented in this study have been deposited in the NCBI GenBank repository and can be found at: https://www.ncbi.nlm.nih.gov/genbank/; OR271589-OR271593.

### Copyright

© The Authors 2023.
