# Supplementary material for: Evolved distal tail protein of skunaviruses facilitates adsorption to exopolysaccharide-encoding lactococci
Source: Microbiome Res Rep. 2023 Jul 18;2(4):26. doi: 10.20517/mrr.2023.29 (PMC10688798; doi:10.20517/mrr.2023.29)
Supplement: Supplementary file 1 [file mrr-2-4-26-SupplementaryMaterials.pdf]

Open Access

Supplementary Material: Evolved distal tail protein of skunaviruses facilitates adsorption to exopolysaccharide-encoding lactococci

Supplementary Table 1. Amino acid identity among evolved Dits in this study.

		Amino Acid Identity (%)											
		Typical								EpsM Variant			
		D4839	D6869	D6875	D6887	D6888	D970	D1113	D6067	D753	D4006	D4842	D5604
Typical	D6869	99.8											
	D6875	97.9	97.6										
	D6887	98.5	98.3	97.6									
	D6888	97.9	97.6	100	97.6								
	D970	91.2	91.4	93.3	91.8	93.3							
	D1113	92.3	92.1	94.4	92.5	94.4	98.9						
	D6067	91.8	91.6	94.0	92.5	94.0	98.9	99.6					
EpsM Variant	D753	61.4	61.4	61.6	61.6	61.6	58.2	58.3	58.3				
	D4006	61.4	61.4	61.6	61.6	61.6	58.2	58.3	58.3	99.8			
	D4842	61.4	61.4	61.6	61.6	61.6	58.2	58.3	58.3	99.7	99.8		
	D5604	61.4	61.4	61.6	61.6	61.6	58.2	58.3	58.3	99.7	99.8	100	
	D2929	58.0	58.0	58.8	58.2	58.8	61.4	61.3	61.3	88.0	87.8	87.8	87.8

Percent pairwise identity of the deduced amino acid sequences of the evolved Dits is shown. Phages that infect strains encoding the typical 6073-like EPS vs. EpsM variant are indicated. Blue box highlights the comparison of Dits belonging to phages infecting strains encoding the typical vs EpsM variant EPS.

© The Author(s) 2021. Open Access This article is licensed under a Creative Commons Attribution 4.0 International License (https://creativecommons.org/licenses/by/4.0/), which permits unrestricted use, sharing, adaptation, distribution and reproduction in any medium or format, for any purpose, even commercially, as long as you give appropriate credit to the original author(s) and the source, provide a link to the Creative Commons license, and indicate if changes were made.

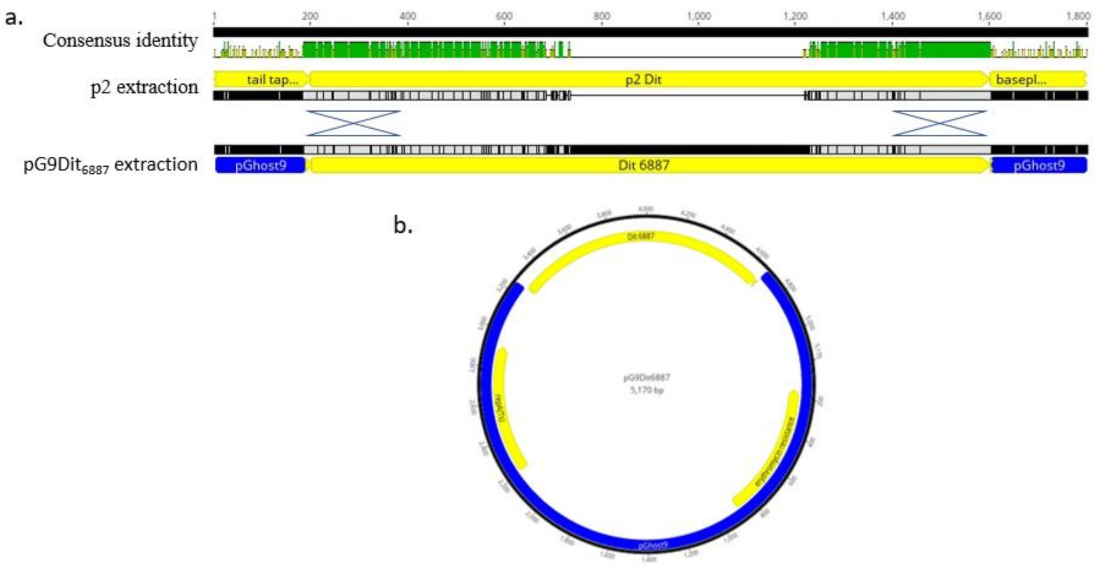

Supplementary Figure 1. Dit exchange by homologous recombination. a. DNA sequence alignment of representative classical *dit* encoded by phage p2 and *dit*$_{6887}$ cloned onto pGhost9, including flanking regions. b. pG9Dit$_{6887}$ vector map. Blue bars indicate pGhost9 sequence. Yellow arrows indicate CDS. In the consensus identity bar, green indicates identity, yellow indicates polymorphism, and red indicates low identity. The sequence homology found at the 5’ and 3’ ends of the *dits* served as regions for homologous recombination.–
